# Rush Medical College

**Published:** 1870-02

**Authors:** 


***Rush Medical College.***

THE Twenty-Seventh Annual Commencement took place Wednesday evening, February 2, 1870, with the following order of exercises:

1. *Prayer*, by Rev. W. H. Van Doren, D.D.; 2. *Music;* 3. *Conferring Degrees;* 4. *Charge to Graduates*, by the President of the Faculty; 5. *Response*, by William R. Aydelott, of the Graduating Class; 6. *Music;* 7. *Valedictory*, by Prof. J. Adams Allen; 8. *Benediction.*

NAMES OF GRADUATES:

Lyman J. Adair, ............Iowa.
Wm. R. Aydelott, .............Ind.
Geo. H. Aurner, ...............Ill.
Thomas J. Adams, ............Ind.
George Theodore Acers, .....Iowa.

D. Bryan Baker, ...............Ill.
C. A. Barnes, .............. ...Ind.
Fred. T. Bicknell, ............Wis.
L. Lafayette Bond, ........ ...Wis.
John Ellison Best, ..............Ill.
Gilbert E. Bridgman, .........Can.
John Bloomingstone, ...........Ill.
Cyril P. Brown, .............Mich.
Albert D. Ballou, .............Wis.
William J. Burns, ............Wis.
David O. Bennett, ............Wis.
Thomas Blakeslee, .............Ill.
William M. Boyd, ..............Ill.

William L. Crowder, ........Iowa.
Wilson C. Carver, ............ Ill.
Paul H. Curtner, ..............Ind.
Thomas Coates, .............Iowa.
James McNab Cassels, .......Cana.
Lafayette W. Case, ..........Iowa.
Howard C. Crist, .............Ill.

Michael J. Donnelly, ........Col'o.
Samuel W. Durant, ............Ill.
Samuel T. Davis, ...............Ill.
Isaac R. Dunning, ...........Mich.
Edward F. Dann, ............Wis.
Daniel L. Dakin, ............Mich.
John W. Dod, ..................Ill.
Jacob R. Dosch, .............Iowa.

Hamilton P. Duffield, ..........Ill.

Richard J. Eaton, ..............Ill.
Milton H. Everett, ..............Ill.
William C. Riehelberger, ......Ind.
Robert S. Edgar, ..............Ill.
Perry M. Evans, ...............Ill.

Abel Ford, Jr., ..............Mich.
William E. Fenwick, .........Mich.
Edward R. Fletcher, ..........Min.
George S. Focht, ............Iowa.
S. Campbell Fenton, ..........Ind.
William Fox, .................Wis.
Benjamin F. Farley, ............Ill.

Augustus H. Guernsey, .......Wis.
John Green, ...................Ill.
George Green, .. ..............Ill.
Strader S. Goldsberry, ........Ind.
Samuel W. Gould, ............Ind.
John W. Goe, ................Wis.
Joseph C. Gifford, .............Ind.
Jesse T. B. Gephart, ..........Kan.
O. G. Given, .................Kan.

William Henry, ................Ill.
Benjamin R. Helms, .........Ind.
Geo. Washington Hudson, .....Ill.
William Harvey, ............C. W.
Frederick C. Hageman, .........Ill.
Marcus M. Hale, ............Ind.
Thomas A. Holman, ...........Ill.

Bishop B. Kelley, .............Neb.
Adrian A. Kitchingman, ......Wis.

Horace R. Littlefield,..........Ill.
August Liljencrantz, .........Wis.
Ledyard Verdine Lewis,......Wis.
Clark Leal,................... Ill.
Benjamin F. LaRue,.... .....Min.
John M. Lester,.............Cana.
Frank L. Lewis,..............Min.
Allen R. Law................Wis.
Lawrence A. Laurason,......Mich.
Stephen W. Lee,..............Ill.

Phineas I. Mulvane,...........Ill.
William L. McLane,.... ......Ill.
Her. Walter Morehouse,........Ill.
Andrew J. Moore, ...........C. W.
George P. Morey,............N. Y.
Pierre L. Monast,..............Ill.
James A. Matthews,.... ......Mo.
Samuel Miller,................Min.
Simon P. Morse,....... ......Ill.
D. H. McFarland,..............Ill.
Albert B. Modesitt,............Ind.
William O. Mendenhall,........Ill.
Henry M. Marvin,...........Mich.
William J Moore,............ Ill.
Charles D. Manning,........Iowa.
Julius A. Morris,............Ohio.
T. Fletcher McFarland,.........Ill.
George B. Noyes,..............Ill.

Oliver C. Ormsby,..........Utah.

Milo Place,....................Ill.
Lewis C. Page,...............Ind.
William H. Palmer,...........Ill.
Francis M. Pickins,...........Ind.
Robert O. Purviance,..........Ill.

Benjamin T. Phillips,.........Wis.
William B. Porter,...........Iowa.
Judson C. Panter,..............Ill.

Charles E. Quire,............Iowa.

James C. Reynolds,..........Wis.
James W. Reeder,............. Ill.
Charles W. Russell,...........Ind.
Walter F. Randolph,...........Ill.

John Wiley Snider,...........Ind.
Zachary T. Stanley,............Ill.
William H. Stewart,.........Ohio.
Theophilus Sprague,...........Ill.
William M. Smith,............Ill.
Henry C. Soule,..............Wis.
James B. Stetson,..............Ill.
H. Watson Smith,............Wis.
Conrad Secrest,...............Ill.
Lewis A. Snyder,.............Ind.
John H. Stewart,...............Ill.
Jacob D. Smith,................Ill.
John T. Scott,................Ind.

John W. Tope,..............Ohio.
Samuel L. Tyner, ............Ind.
J. Austin Thompson,........Iowa.
William Todd,................Cal.

Leonard P. Woodworth, .. ..Wis.
Delinso A. Walden,............Ill.
Charles A. Wilcox,............Ill.
John C. Webster,.............Ind.
Albert Wilgus,................Ind.
John C. Waite,...............Ind.
Gideon A. Weed,.... .......Cal.

### Ad Eundem Degree.

Richard H. Plummer, M.D.,...Cal.

Louis Stoskof, M.D.,...........Ill.

David Dodge, M.D..............Ill.

J. F. Grimes, M.D.,..........Iowa.

### Honorary Degree.

Andrew McFarland, M.D.,...................................Ill.

The spacious lecture-room was crowded, and great enthusiasm was manifested, culminating in repeated and tempestuous rounds of applause, when Dr. J. R. Dosch stepped forward and presented the veteran Prof. Freer, on behalf of the graduating class, with an elegant gold-headed cane. Dr. Dosch's remarks were in excellent taste, and Prof. Freer, although taken wholly by surprise, responded with feeling and eloquence.

We chronicle this Commencement as one of the most pleasant and auspicious in the history of the College.

